# Natural History, Neuroradiological Workup, and Management Options of Chronic Atlantoaxial Rotatory Fixation Caused by Drug-Induced Cervical Dystonia

**DOI:** 10.1155/2021/6683268

**Published:** 2021-03-03

**Authors:** Yuki Ishikawa, So Kato, Mario Ganau, Shima Hirai, Yasushi Oshima, Sakae Tanaka

**Affiliations:** ^1^Department of Orthopaedic Surgery, The University of Tokyo, 113-8655, 7-3-1, Hongo, Bunkyo-ku, Tokyo, Japan; ^2^Department of Neurosurgery, Oxford University Hospitals NHS Foundation Trust, Oxford, UK

## Abstract

Atlantoaxial rotatory fixation (AARF) resulting from drug-induced cervical dystonia (DICD) represents an extremely rare complication of antipsychotic treatment, requiring a comprehensive assessment of pharmacologic therapy and timely radiologic workup. We report a chronic case of Fielding type I, Pang type I AARF secondary to schizophrenia treatment in a 16-year-old girl, along with a review of the literature on the management challenges posed in this condition. In this scenario, torticollis may just represent the tip of the iceberg, and only an effective multidisciplinary approach increases the chances of satisfactory correction with closed reduction, hence avoiding the burden of more invasive treatment options.

## 1. Introduction

The rotatory subluxation of the atlantoaxial joint, also known as atlantoaxial rotatory fixation (AARF), is a frequent cause of torticollis in children [1], which was firstly reported by Corner in 1907 [2]. A thorough clinical description of this condition was provided by Wortzman and Dewar in 1968 [3]; nonetheless, the term AARF was introduced only by Fielding and Hawkins who also classified AARF into 4 types (Table 1) in 1977 [4]. Over the years, this condition has been described as atlantoaxial rotatory subluxation [3, 5, 6], dislocation [7], or displacement [8], making the pertinent literature fairly heterogeneous until the seminal work from Pang led to the introduction and diffusion of a more modern classification and management algorithm (Table 1) [9].

**Figure 1:**
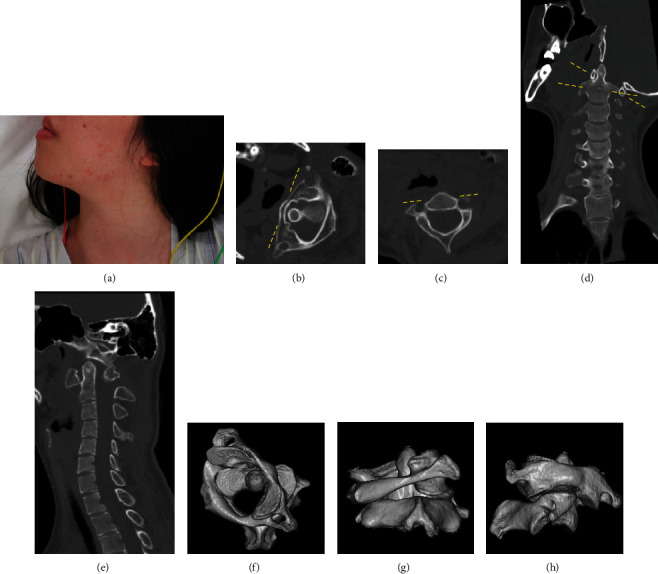
Patient position before reduction (a) and CT of the cervical spine on admission (b–h). (b) Axial view of C1, (c) axial view of C2, (d) coronal view, (e) sagittal view, and (f–h) three-dimensional reconstruction. The C1-C2 rotatory angle was 60 degrees.

Patients with AARF present with neck pain, limited neck motion, and the so-called cock-robin posture, where the head is rotated to one side and on lateral flexion contralaterally (Figure 1(a)); the most common causes are trauma and infection, rheumatoid arthritis, head and neck surgery, and congenital connective disorders [10, 11].

Rarely, cervical dystonia has been linked to AARF [12–14]. Although it is known that antipsychotics sometimes induce cervical dystonia [15, 16], there have been no reports of AARF caused by drug-induced cervical dystonia (DICD). We report such a rare presentation during schizophrenia treatment, enriched by a review of the literature.

## 2. Case Report

### 2.1. Clinical History

A 16-year-old girl with recent diagnosis of schizophrenia was prescribed treatment with antipsychotics. After few months, she started to have neck pain and torticollis and two years later was diagnosed with antipsychotic-related tardive DICD. An antiparkinsonian agent was started, and botulinum toxin was injected twice, yielding minimal improvement. After a year of unsuccessful treatment, she was hospitalized for further treatment and was referred to our department for evaluation of the torticollis.

### 2.2. Examination and Diagnostic Workup

Due to the uncontrolled schizophrenia, she had delusions, hallucinations, and disorganized thinking and behaviors. Although communication was very difficult, the patient was identified to be suffering from persistent neck pain and difficulties with eating, swallowing, and gaze. She was fixed in a typical cock-robin posture; the neck was twisted to the right and flexed to the left (Figure 1(a)). She had no neurological deficits.

Computed tomography (CT) revealed Fielding type I and chronic Pang type I AARF [4, 17] (Figures 1(b)–1(h)). The atlas was rotated 60 degrees to the right in the axial view (Figures 1(b) and 1(c)). The lateral inclination of the atlas was 25 degrees in the coronal view (Figure 1(d)). The atlantodental interval (ADI) was 2 mm in the sagittal view (Figure 1(e)). In 3D reconstruction images, the C1-C2 joint was locked, although C2 facet deformity was not confirmed (Figures 1(f)–1(h)).

### 2.3. Treatment Course

**Figure 2:**
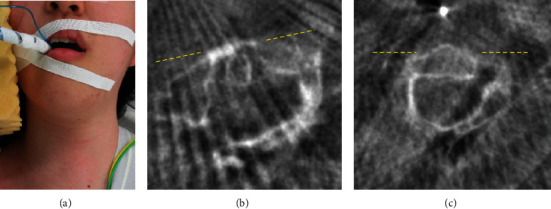
Patient's neck position (a) and CT after the first reduction (b, c). (b) Axial view of C1 and (c) axial view of C2. The C1-C2 rotatory angle decreased to 15 degrees.

Considering her mental condition, continuous traction, halo vest, and surgical intervention were not deemed appropriate in the first instance, due to safety concerns. She was mentally unstable and had a risk of taking unpredictable movements; hence, the consensus from multidisciplinary team (MDT) discussion was to perform only closed manual reduction followed by immobilization with the Philadelphia collar. Closed manual reduction was conducted under general anesthesia and fluoroscopy (Arcadis Orbic 3D, Siemens Healthineers) in the operation room. Neck alignment appeared normalized in the neutral position (Figure 2(a)), and reposition of the atlas was confirmed with fluoroscopy (Figures 2(b) and 2(c)). The patient was placed in a Philadelphia collar fixation immediately.

### 2.4. Outcome

**Figure 3:**
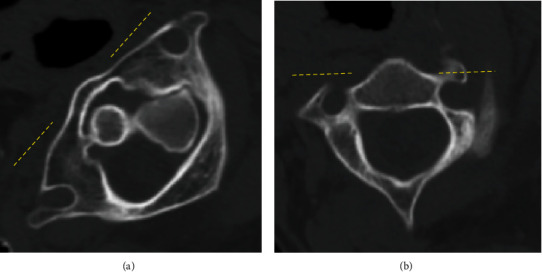
CT showing recurrence of AARF. (a) Axial view of C1 and (b) axial view of C2. The rotatory angle increased to 45 degrees.

**Figure 4:**
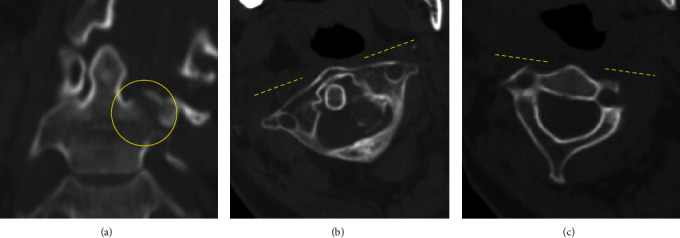
CT after the second reduction, revealing the fracture of the C2 lateral mass (yellow circle). (a) Axial view of C1, (b) axial view of C2, and (c) coronal view. Atlantoaxial rotatory subluxation remained, although the C1-C2 rotatory angle decreased to 30 degrees.

**Figure 5:**
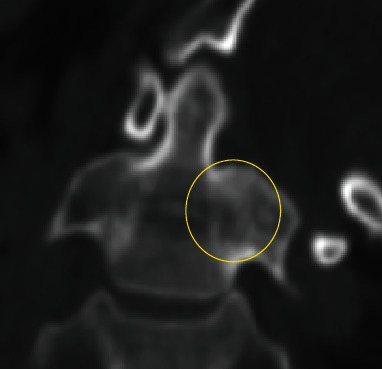
Bony union was obtained 2 months later (yellow circle).

Unfortunately, few days later, torticollis recurred despite good compliance with the Philadelphia collar. The atlas was rotated 45 degrees to the right from the neutral position (Figure 3). The strategy adopted included an immediate attempt to achieve reduction again under general anesthesia; nonetheless, this also led to an iatrogenic fracture of C2 lateral mass (Figure 4(a)) and atlantoaxial rotatory subluxation remained (Figures 4(b) and 4(c)). Family discussion was held, and surgical option to minimize the chances of recurrence or pseudoarthrosis was abandoned given the high perioperative risks. The final decision was therefore to continue conservative treatment with the Philadelphia collar and carry out a close clinical and radiological monitoring. Bony union was obtained 2 months later (Figure 5), and during follow-up, the patient demonstrated a consistent improvement of neck pain, range of neck motion, and activities of daily living (ADL); therefore, no further spinal treatment was needed. Five months after the fracture, range of neck motion had improved to 60 degrees, which was considered to be a good result, given that the C1-C2 joint was already completely “locked” before treatments.

## 3. Discussion

There have been no previous reports of AARF with drug-induced cervical dystonia. Tardive dystonia is known as one of the side effects of antipsychotics, and the most frequent type is cervical dystonia [16, 18]. The first choice of treatments of cervical dystonia is oral medications such as trihexyphenidyl, benzodiazepines, or tetrabenazine. Other choices are botulinum injection and deep brain stimulation [19–22]. Although the mechanisms underlying AARF in patients with cervical dystonia are not clearly understood, it is assumed that the contraction of the sternocleidomastoid muscle causes rotatory subluxation of the atlas and the axis.

Pang's classification, which further groups patients into acute, subacute, and chronic, is particularly useful to guide the treatment strategy. Whereas acute (<1 month) and subacute (>1-<3 months) AARF can usually be treated by nonoperative treatment such as closed manipulation or cervical traction followed by bracing [10, 23], the treatment strategy for chronic AARF (lasting >3 months) is controversial. Halter traction and skull caliper traction are recommended as the initial treatment in the algorithm by Pang and Li [23]. Ishii et al. reported that closed manipulation followed by halo fixation is an effective treatment. Surgical treatments are recommended for cases with C1-2 bony union, irreducible or recurrent subluxation, and Fielding type 4 [10]. Treatment options of AARF are shown in Table 2 [10, 24–28].

The complexity of treating patients with uncontrolled schizophrenia relates to their tendency toward unpredictable movements and higher rates of postoperative complications [29, 30]. Any invasive treatments, such as traction and surgery, are therefore not recommended. When treating this challenging category of patients, it is important to ensure a comprehensive diagnostic workup and start closed reduction at an early stage, before traction, halo fixation, or surgery becomes a necessary step. Optimization of medical treatment and immobilization in the hard collar may prove safer and relatively effective; nonetheless, a close clinical and radiological monitoring is recommended due to the inherently high risk of failure.

## 4. Conclusion

AARF resulting from DICD is a rare complication of antipsychotic treatment, requiring a comprehensive assessment of pharmacologic therapy and timely radiologic workup. In this scenario, torticollis may just represent the tip of the iceberg, and only an effective multidisciplinary management can increase the chances of a satisfactory correction with closed reduction, hence avoiding the burden of more invasive treatment options.

## Figures and Tables

**Table 1:** AARF classifications.

Fielding and Hawkins AARF classification [4]	
Type 1	Rotatory fixation with no anterior displacement
Type 2	Rotatory fixation with anterior displacement of 3 to 5 millimeters
Type 3	Rotatory fixation with anterior displacement of more than 5 millimeters
Type 4	Rotatory fixation with posterior displacement

Pang AARF classification [9, 17]	
Type 1	AARF patients show essentially unaltered (“locked”) C1-C2 coupling regardless of corrective counterrotation
Type 2	AARF patients show reduction of the C1-C2 separation angle with forced correction, but C1 cannot be made to cross C2
Type 3	AARF patients show C1-C2 crossover but only when the head is cranked far to the opposite side

**Table 2:** Treatment options for patients with AARF, with respect to their specific effectiveness and pros and cons [10, 24–28].

Treatment options		Effectiveness	Pros	Cons
Conservative strategies	Anti-inflammatory drugs	Limited	No mechanical invasiveness	Gastrointestinal bleeding, allergic reactions
	Cervical collar	Limited	Minimum invasiveness	Skin decubitus
	Halter traction Glisson traction	Limited	Low invasiveness	Difficult airways access and feeding
	Skull traction	Intermediate	Intermediate invasiveness	Pin site infection
	Closed reduction under general anesthesia plus halo vest immobilization	High	Effective, even to chronic AARF	Pin site infection

Surgical strategies	Posterior approaches for C1-C2 fixation (various techniques: wiring, hooks, Magerl transarticular screws, and Harms technique with C1 lateral mass and C2 pars/pedicle/translaminar screws)	Very high	Effective in irreducible or recurrent subluxation, Fielding type 4 AARF	Damage to the vertebral artery, C2 nerve root ganglion, dural tears with cerebrospinal fluid leakage
	Anterior approaches for C1-C2 fixation (various routes including lateral neck and transoral; various techniques: transarticular screws and plating)	Very high	Effective in cases with C1-C2 bony union	Damage to the hypoglossal nerve, internal carotid artery, and soft viscera Need for nasogastric feeding
